# Vector Competence of *Eucampsipoda africana* (Diptera: Nycteribiidae) for Marburg Virus Transmission in *Rousettus aegyptiacus* (Chiroptera: Pteropodidae)

**DOI:** 10.3390/v13112226

**Published:** 2021-11-04

**Authors:** Janusz T. Pawęska, Petrus Jansen van Vuren, Nadia Storm, Wanda Markotter, Alan Kemp

**Affiliations:** 1 Centre for Emerging Zoonotic and Parasitic Diseases, National Institute for Communicable Diseases of the National Health Laboratory Service, Sandringham 2131, South Africa; Petrus.Jansenvanvuren@csiro.au (P.J.v.V.); nstorm@bu.edu (N.S.); alank@nicd.ac.za (A.K.); 2 Centre for Viral Zoonoses, Department of Medical Virology, Faculty of Health Sciences, University of Pretoria, Pretoria 0001, South Africa; Wanda.Markotter@up.ac.za; 3 School of Pathology, Faculty of Health Sciences, University of Witwatersrand, Johannesburg 2050, South Africa; 4 Australian Centre for Disease Preparedness, CSIRO Health & Biosecurity, Geelong, VIC 3220, Australia; 5 Department of Microbiology, School of Medicine, Boston University, Boston, MA 02118, USA

**Keywords:** Marburg virus, bat flies, vector competence, field infection rate, transmission, Egyptian rousette bat

## Abstract

This study aimed to determine the vector competence of bat-associated nycteribiid flies (*Eucamsipoda africana*) for Marburg virus (MARV) in the Egyptian Rousette Bat (ERB), *Rousettus aegyptiacus*. In flies fed on subcutaneously infected ERBs and tested from 3 to 43 days post infection (dpi), MARV was detected only in those that took blood during the peak of viremia, 5–7 dpi. Seroconversion did not occur in control bats in contact with MARV-infected bats infested with bat flies up to 43 days post exposure. In flies inoculated intra-coelomically with MARV and tested on days 0–29 post inoculation, only those assayed on day 0 and day 7 after inoculation were positive by q-RT-PCR, but the virus concentration was consistent with that of the inoculum. Bats remained MARV-seronegative up to 38 days after infestation and exposure to inoculated flies. The first filial generation pupae and flies collected at different times during the experiments were all negative by q-RT-PCR. Of 1693 nycteribiid flies collected from a wild ERB colony in Mahune Cave, South Africa where the enzootic transmission of MARV occurs, only one (0.06%) tested positive for the presence of MARV RNA. Our findings seem to demonstrate that bat flies do not play a significant role in the transmission and enzootic maintenance of MARV. However, ERBs eat nycteribiid flies; thus, the mechanical transmission of the virus through the exposure of damaged mucous membranes and/or skin to flies engorged with contaminated blood cannot be ruled out.

## 1. Introduction

Marburg virus (MARV), species *Marburg marburgvirus* (*Filoviridae*) [1], causes sporadic but often fatal Marburg Virus Disease (MVD) in humans [2]. To date, fourteen outbreaks of MVD have been reported, most occurring in sub-Saharan Africa [2,3], but a single MVD case was reported in August 2021 from Guinea, West Africa [4]. Evidence of marburgvirus circulation was also reported from countries where MVD cases have not been recorded [5,6,7]. The first recognised outbreak of MVD in Africa occurred in 1975 [8] with the largest and most deadly reported from Angola in 2004–2005, with a case-fatality rate of 90% [9].

In the past two decades, there has been significant progress in studies of bats as reservoirs of diverse pathogens of public health importance [10,11], including filoviruses [12,13,14], coronaviruses [15,16,17,18,19,20], paramyxoviruses [19,21,22,23,24], and lyssaviruses [25,26]. The results of ecological, epidemiological, and experimental studies implicate the ERB as the prime reservoir host for marburgviruses [13,14,27,28,29,30,31,32,33].The roosting habitats of the Egyptian Rousette Bat (ERB), *Rousettus aegyptiacus*, including caves and mines, have been associated with infection of humans with MARV [13,14,27,34,35,36,37,38]. 

The identification of natural entry and exit portals for marburgviruses in ERBs and, consequently, mechanisms involved in the maintenance of marburgviruses in bat colonies remain elusive. Experimental studies demonstrated that the infection of ERBs with MARV results in viremia and replication of the virus in multiple tissues, including organs compatible with viral shedding (salivary gland, kidney, bladder, intestines, reproductive tract) [28,29,30,31,32,33]. It appears that the clearance of MARV from the salivary glands of experimentally infected ERBs is slower than from other tissues [30,31,32].

Both oral and faecal shedding of MARV has been demonstrated in wild caught and experimentally infected captive-bred ERBs [14,30,31,32,39]. Oral shedding resulting in contamination of the oral cavity might contribute to MARV transmission via biting. Biting is observed among ERBs, with juveniles being the most adversely affected, as they are bitten by older bats displaying dominance. Potential faecal–oral transmission of marburgviruses highlights the risk of environmental contamination, and ERB roosting sites might be a source of virus spillover to humans. Roosting behaviour, including clustering into close and dense groups, auto-, and allo-grooming result in direct physical contact that would support faecal–oral transmission of marburgviruses in bats. Moreover, biting by hematophagous ectoparasites might result in inoculation of wounds with contaminated saliva and faeces or exposure to contaminated fomites. However, it remains unknown if faecal–oral transmission represents a natural portal of entry for marburgviruses, to what extent viral shedding patterns found in captive-bred ERBs can be extrapolated to natural settings, and whether this mode of transmission represents a sustainable mechanism for the enzootic perpetuation of marburgviruses.

Limited experimental studies of horizontal transmission of MARV between experimentally infected and naïve in-contact ERBs have yielded intriguing results [29,32]. In a study by Paweska et al. [29], neither seroconversion nor viremia could be demonstrated in any of 14 in-contact susceptible ERBs up to 42 days after exposure to 22 infected bats by subcutaneous (SC) MARV inoculation, despite demonstrable shedding of the virus via saliva, faeces, and urine. A similar MARV transmission experiment was conducted by Schuh et al. [32] for a period of 9 months. In this study, SC-infected donor bats were cohoused for 56 days directly with naïve contact bats or placed over cages of additional naïve bats, thus potentially exposing them to infectious urine, faeces, and fruit spats. Thereafter, all bats were grouped and housed together for an additional 7 months. MARV shedding in saliva or urine was detected in 11 of 12 inoculated bats between 5 and 19 days post infection (PI). Interestingly, MARV viremia was only detected in three naïve contact bats at 7 months PI, one of which tested positive by oral swab. Two of these three bats seroconverted by 8 months PI, and by 9 months PI, nine (37.5%) of the 24 naïve contacts bats had seroconverted. While it is rather puzzling why less than half of the naïve contact bats seroconverted, this study suggests a prolonged incubation period (up to 7–8 months) in in-contact infected bats after extended exposure to MARV-infected donor bats and their bodily secretions. The mechanism by which the virus could survive in in-contact infected bats during this lengthy incubation remains unclear. The establishment of persistent infection in immune-privileged sites, at least in some proportion of MARV-infected ERBs, might be one possibility.

MARV has been shown to be present in the male and female reproductive tracts of experimentally infected ERBs [29]. Both MARV and *Zaire ebolavirus* have been isolated from human seminal fluids months after the patient’s full clinical recovery [40,41,42,43], which may serve as additional evidence for a sexual route of MARV transmission in ERBs. During the 2014–2016 West African Ebola Virus Disease (EVD) epidemic, sporadic transmission events resulted in the initiation of new chains of human-to-human transmission. Multiple reports suggest that these re-emergences were linked to persistent EBOV infections and included sexual transmission from EVD survivors [44,45,46,47,48,49]. Ebola virus RNA has also been detected in other immunologically privileged sites in humans and non-human primates such as the central nervous system and eyes [50,51,52,53,54].

The role of ectoparasites in filovirus transmission has been postulated [55]. While it appears that MARV can be horizontally transmitted among ERBs under experimental conditions in the absence of arthropods [32], the potential for arthropod involvement as an intermediate host has never been definitively disproven.

Hematophagous arthropods are responsible for the biological or mechanical transmission of numerous human and animal pathogens such as viruses, bacteria, protozoa, and helminths that can be acquired through ingestion or by contact [56,57,58]. Bats are parasitised by a number of ectoparasites, including mites, bat flies, ticks, and fleas [59]. One of aspects of the host–pathogens relationship is the role of ectoparasites as reservoirs and vectors of zoonotic diseases, including hematophagous bat flies of families Streblidae and Nycteribiidae. Members of these two families are highly host-specific obligate ectoparasites of bats [59,60,61,62] and are potentially capable of mechanical or biological pathogen transmission. *Bartonella* and *Rickettsia* [63,64,65], viruses from the Rhabdoviridae family [66,67], and protozoans including the hematoparasite *Polychromophilus* [68] have been detected in bat flies.

Kunz and Hofmann [69] successfully propagated MARV in experimentally inoculated *Aedes aegypti* (Diptera: Culicidae), but not in *Anopheles maculipennis* and castor bean ticks (*Ixodes ricinus*). Different arthropods species have been tested as part of ecological investigations in past filovirus outbreaks, without any positive results, but these studies excluded bat flies [42,70,71,72]. Bat flies (Diptera: Nycteribiidae) and argasid ticks, *Ornithodoros faini*, collected directly from bats and off the walls at the roost sites in Python Cave where active marburgvirus infection was occurring, tested negative for marburgviruses [14,38], but their number tested was too low to reasonably determine that these ectoparasites are not involved in the marburgvirus transmission. Schuh et al. [73] presented more evidence after testing 3125 *O. faini* ticks collected from the walls of Python Cave, where ERBs were roosting. All were negative for marburgviruses, indicating that these ticks do not act as replication or mechanical vectors for marburgviruses in ERB populations.

Results of recent studies in South Africa demonstrate enzootic and very efficient marburgvirus circulation in ERBs roosting at Matlapitsi Cave, which is located in the rural area of the Limpopo province of the country [39,74]. This colony is heavily infested by hematophagous ectoparasites throughout the year and particularly by nycteribiid flies *Eucampsipoda africana* (Diptera: Nycteribiidae). A novel fusogenic orthoreovirus and a novel orthobunyavirus have been isolated from *E. africana* collected from ERBs in this cave [75,76].

Knowledge on natural filovirus transmission would help to understand the mechanisms involved in zoonotic spillover and aid public health control measures. The development of viremia observed in ERBs after experimental subcutaneous infection with MARV [28,29,30,31,32,33] suggests that blood-sucking ectoparasites might be involved in the maintenance and perpetuation of the virus. Our study aimed to determine the vector competence of *E. africana* for the replication and transmission of MARV to ERBs. In addition, we tested nycteribiid flies collected from ERBs in Matlapitsi Cave to determine the field infection rate for marburgvirus.

## 2. Materials and Methods

### 2.1. Description and Ecology of Matlapitsi Cave

**Figure 1:**
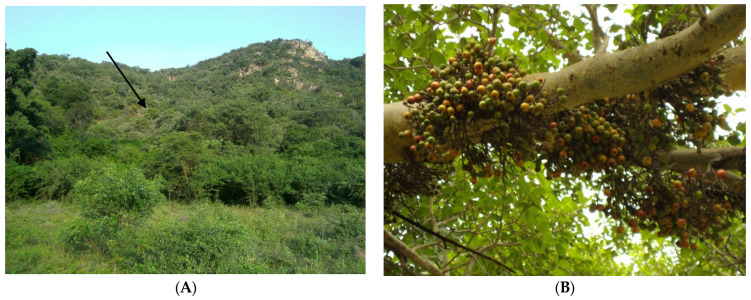
Location of entry to Matlapitsi cave (indicated by arrow) inhabiting R. *aegyptiacus* colony, Matlapitsi Valley, Limpopo province, South Africa (**A**); Cape fig tree with fruits, which is a preferred food for R. *aegyptiacus* (**B**).

The Matlapitsi cave (previously also called Mahune cave) [39,77] is located in the indigenous flora of the Matlapitsi Valley (24°1′ S, 30°10′ E) on the north-eastern slope of the Wolkberg Mountain range, bordering the Lekgalameetse Nature Reserve in the Limpopo province, South Africa. The cave is situated in close proximity to the semi-rural community of Fertilis, which is hidden by the indigenous flora (Figure 1A). It is inhabited by a large ERB population, which represents a maternity colony. In the Matlapitsi Valley, figs form the basis for this cave-dweller bats’ diet, in particular *Ficus sycamorus* (Figure 1B), but *F. petersii* and *F. sansibarica* and other fruits are also eaten at certain times of the year [77].

**Figure 2:**
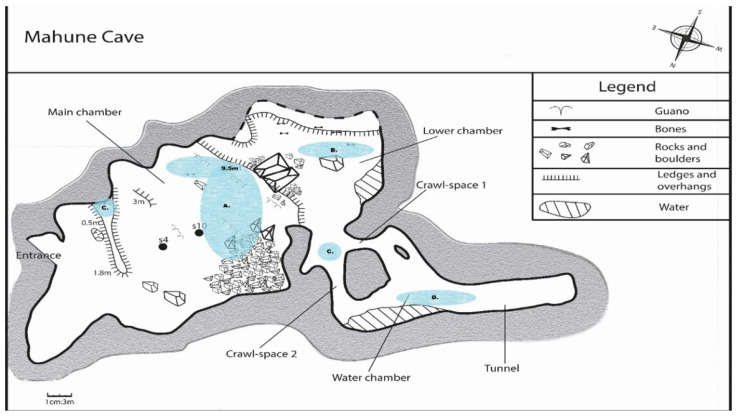
Transectional view of Matlapitsi cave system at a scale of 1 cm:3 m: Shaded area A. indicates the roosting area of *Rousettus aegyptiacus* in the main chamber of the cave. Shaded areas B. indicate the areas where *Miniopterus natalensis*, *Myotis tricolor*, and *Hipposideros caffer* roost when present. Shaded areas C. indicate the only areas that *Nycteris thebaica* and *Rhinolophus* spp. have been observed roosting.

The approximately 5 m arched opening of the cave leads into the main chamber of about 6000 m^3^. The cave floor slopes away steeply from the entry, leaving the roof some 7 m above, whilst the cave widens to almost 34 m and extends 28 m horizontally into the mountain. Proceeding 20 m south-west into the cave, an aggregation of 2–3 m^3^ boulders towers towards the ceiling, where the ERBs roost across the creviced roof. The back corner of the cave drops 9.5 m to a smaller second chamber of about 700 m^3^, 15 m at its widest, 4.5 m at its highest, and extending another 12 m south by southwest into the rock. This second chamber appears to reach the water table, with a yearlong clear still pool and is frequented by the Natal long-fingered bat (*Miniopterus natalensis*). Branching off northwest from the second chamber is a 6 m^3^ alcove, which hosts two small crawl spaces that lead to the third chamber. This last chamber is about 9 m long with the roof only 3.8 overhead and extends west by southwest before extending into a tunnel almost 2 m in diameter that extends for another 12.7 m before terminating abruptly in a solid rock fall (Figure 2).

The Matlapitsi ERB population represents a nursery colony with fluctuating numbers during the year. The population density increases during the hot, rainy season (October–February) when food is the most abundant, reaches a maximum during March–April, and declines in numbers from June to July when food availability is low [77]. Apart from ERB and *M. natalensis*, the following bat species are present at Matlapitsi cave: short-eared Trident bat (*Cloeotis percivali*), Temminck’s hairy bat (*Myotis tricolor*), Hildebrandt’s horseshoe bat (*Rhinolophus hildebrandtii*), the Bushveld horseshoe bat (*Rhinolophus Simulator*), Blasius’s horseshoe bat (*Rhinolophus blasii*), Darling’s horseshoe bat (*Rhinolophus darlingi*), Geoffroy’s horseshoe bat (*Rhinolophus clivosus*), Sundevall’s roundleaf bat/Sundevall’s leaf-nosed bat (*Hipposideros caffer*), and the Egyptian slit-faced bat (*Nycteris thebaica*). The ERBs permanently and almost exclusively inhabit the main chamber of the cave. The second and third chamber is frequented by *M. natalensis* throughout the year, but their numbers are particularly high (several thousand) from October until December. *M. natalensis* is accompanied by smaller numbers of *M. tricolor* and *H. caffer.* The other year-long inhabitants, *N. thebaica* and *Rhinolophus* spp., have been observed roosting in both the alcove and in the small crawl spaces of the cave entrance. Other animal species observed around the cave include Cape porcupine *(Hystrix africaeaustralis*), vervet monkey (*Chlorocebus pygerythrus*), the common genet (*Genetta genetta*), baboons (*Papioursinus*), black-backed jackals (*Canis mesomelas*), and domesticated goats (*Capra aegagrus hircus*). Black mamba (*Dendroaspis polylepis)* has been seen near the cave entrance, whilst a pair of resident African rock pythons (*Python sebae*) have been observed catching and eating bats.

During monthly visits to the cave from June 2012 to July 2018, infestation of bats by different ectoparasites, including mites, ticks, fleas, and bat flies of families Streblidae and Nycteribiidae was noted throughout the year. Bat flies *E. africana* (family Nycterbiidae) have been found predominantly on ERB and *N. thebaica* bats. The identification and confirmation of bat fly species infesting ERBs bats was done using a morphological key [78] and by the amplification of the cytochrome c oxidase subunit I (COI) gene using barcoding primers as previously described [75].

The annual average temperature recorded at the roost of the cave from 2014 to 2018 was 21.8 °C with a minimum of 18.5 °C and maximum of 34.6 °C, and the annual average relative humidity (RH) was 86.8% with minimum of 48.4% and maximum of 98.6%. Temperature and RH were recorded using data loggers LogTag^®^ HAXO-8 Humidity & Temperature Recorder (LogTag^®^ Recorders, Cape Town, South Africa).

### 2.2. Collection of Bat Flies from Egyptian Rousette Bats

**Figure 3:**
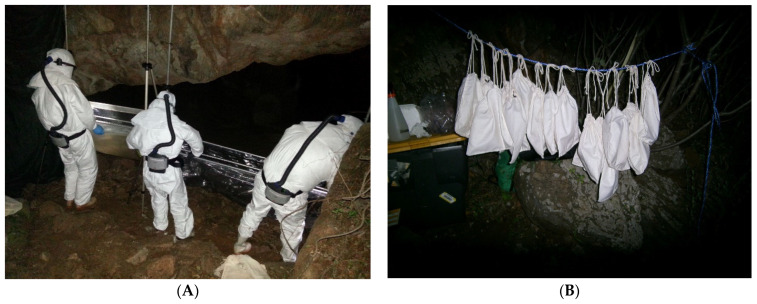
Collecting of bats trapped in harp traps by operators dressed in BSL3 personal protective equipment at the entrance to Matlapitsi cave, Matlapitsi Valley, Limpopo province, South Africa (**A**); Captured R. *aegyptiacus* placed in individual cotton bags until sampling (**B**).

ERBs were captured at Matlapitsi Cave, using two harp traps (G5 Bat Trap, Bat Conservation and Management, Carlisle, PA, USA) (Figure 2A) [79] across the cave entrance, with staff wearing personal protective equipment including powered air-purifying respirators and coveralls (Figure 3A) typically worn in biosafety level 3 (BSL3) laboratory environments [14]. All staff involved in bat trapping and sampling had been vaccinated against rabies previously. The collection bag of each harp trap was checked every 10–20 min; trapped bats were removed and temporally placed in individual cotton bags prior to sampling (Figure 2B).

**Figure 4:**
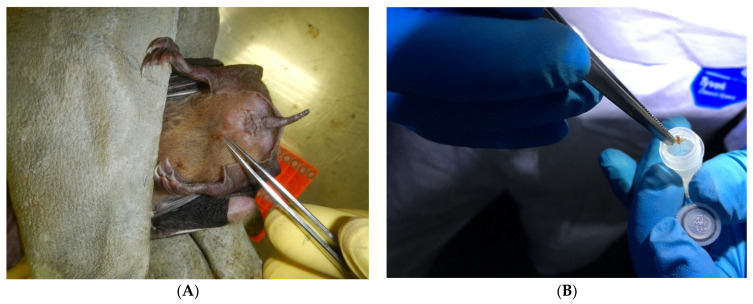
Field collection of bat fly *Eucampsipoda africana* (family Nycterbiidae) from *R. aegyptiacus* bats (**A**) into a microtube for transportation to containment facility (**B**).

For laboratory testing, bat flies were collected from ERBs (Figure 4A) into 2 mL cryotubes (Figure 4B) containing 0.3 mL of Dulbecco’s modified Eagle’s medium (DMEM, Lonza, Basel, Switzerland). Samples were snap frozen using a vapour phase liquid nitrogen field storage container and transported to the biosafety level four laboratory (BSL4) at the National Institute for Communicable Diseases (NICD) of the National Health Laboratory Service (NICD-NHLS) in Johannesburg for further processing and testing. Collection of bat flies for laboratory testing was done from bats trapped regularly from June 2012 to July 2018 as a part of a biosurveillance project on zoonotic pathogens harboured by South African bats. The trapping and sampling of bats was usually done over two consecutive nights, from 19:00 to 24:00.

Our pilot experiment has demonstrated that to ensure the maximum survival of bat flies, the time from collection at Mahune cave to the time of their release on captive-bred bats in BSL4, should not be longer than 10–12 h. Therefore, to shorten the time needed for collection of the required number of flies for experimental study, they were collected at the peak of ERBs emergence from the cave, usually from 19:30 to 21:00. Flies were placed into 2 mL cryotubes containing a small piece of wetted paper towel and transported in microtube storage boxes as soon as feasible from the Matlapitsi cave site to NICD-NHLS BSL4 (about 5 h drive) for further handling.

### 2.3. Infestation Rate of Egyptian Rousette Bats by Bat Flies

The bat fly infestation rate of ERBs roosting at Matlapitsi cave was calculated based on two collections made in November 2016 using trapping and sampling procedures as described in Section 2.2.

### 2.4. Marburg Virus Infection Rate in Bat Flies

A total of 1653 bat flies collected from ERBs roosting at Matlapitsi cave between June 2012 and July 2018 were processed for laboratory testing. Bat flies were transferred into 2 mL grinding tubes and homogenised at 30 Hz for eight minutes using a Tissuelyzer II (Qiagen, Hilden, Germany) and 5 mm stainless steel beads (Qiagen). Cellular debris was removed by centrifugation at 14,000× *g* for 3 min, and the supernatant was used for virus isolation and detection of MARV RNA as previously described [28,75].

### 2.5. Experimental Animals

**Figure 5:**
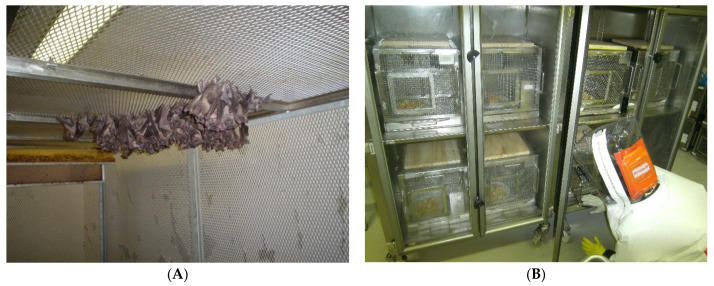
*R. aegyptiacus* colony in flight cage (**A**). Negative pressure HEPA-filtered animal isolators (Techniplast, West Chester, PA, USA) with custom designed stainless steel cages for housing bats in BSL4, NICD-NHLS, Johannesburg, South Africa; untreated wooden planks placed on the celling of cages serving as a roosting substrate for laying bat fly pupae (**B**).

The source for ERBs bats and trapping procedures were the same as described previously for the experimental infection study with MARV [29]. Wild caught bats were transported to a BSL3 animal quarantine facility where they were kept for 6 weeks in custom-designed stainless steel cages. Bats were housed in groups of 5–6 animals per cage (Figure 5A), which were placed in negative pressure HEPA-filtered cabinets (Techniplast, West Chester, PA, USA) and fed as previously described [28,29]. Bats were tested at 3-weekly intervals to confirm that they remained serologically negative for antibodies against filoviruses or rabies-related lyssaviruses prior to moving to a flight cage for housing (Figure 5B). Colony conditions were maintained as previously described [28,29].

All work with infectious virus, inoculated animals, and bat flies was conducted at the Centre for Emerging and Zoonotic Diseases, NICD-NHLS, Sandringham, South Africa in a BSL4 laboratory. Bats were transported from the flight cage (Figure 5A) to BSL4 and housed in the same cages, and animal isolators were as used in BSL3 during a quarantine period, except for having an untreated wooden plank (pine wood) placed on the celling of the cage, which served as a roosting substrate for depositing pupas by bat flies (Figure 5B). Bats were acclimatised to the BSL4 environment for one week before the experimental procedures started, were fed fresh tropical fruits and provided with fresh water *ad libitum*, and were monitored daily for the development of clinical signs as well as for food intake.

Husbandry procedures and environmental conditions were identical to those previously described [28] with some modifications. To enhance the RH, animal isolators were modified to allow for an intake of mist produced by humidifiers (Electra, Johannesburg, South Africa). A single humidifier enclosed in a custom-made housing was placed on the celling of the Techniplast cabinet in such a way that the discharged steam could be directed by cabinet vertical airflow into the interior of the isolator (technical modification of Techniplast cabinet for housing a humidifier is available on request). Temperature and RH inside animal isolators were monitored using the same data loggers as those used to take temperature and RH measurements in Matlapitsi cave.

### 2.6. Virus Stock

The SPU 148/99/1 isolate of MARV (second passage in Vero cells) isolated from the serum of a patient who contracted a fatal MVD disease in 1999 in Watsa, Democratic Republic of Congo was used for the subcutaneous (SC) inoculation of bats as described before [29] and for intra-coelomic (IC) inoculation of bat flies.

### 2.7. Experimental Infections

Experiment I: The aim of this experiment was twofold: (1) to investigate the oral susceptibility of bat flies to MARV following feeding on viremic bats, (2) to attempt the transmission of MARV by bat flies from viremic bats to in-contact MARV-seronegative control bats. A week before any experimental procedures started, 35 MARV-seronegative bats, aged 8–12 months, were moved from a flight cage in a BSL3 animal facility to a BSL4 animal containment room. After one week of acclimatisation in BSL4, 27 ERBs were inoculated SC with 100 µL of tissue culture supernatant containing 10^5.3^ mL TCID_50_ of MARV, and 8 ERBs were mock inoculated SC with 100 µL of Eagle’s minimal essential medium (EMEM).

**Figure 6:**
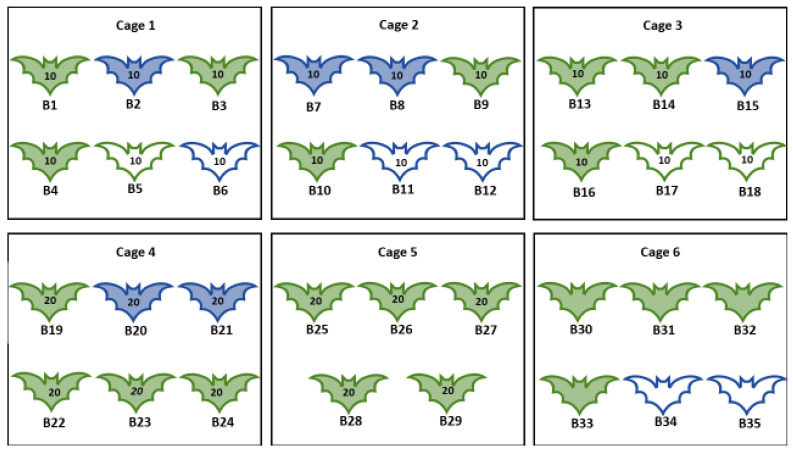
Experiment I. Number, sex, MARV-inoculation status of bats, and the number of bat flies released on Egyptian rousette bats. B = Bat identification number 1–35; Green outline = Female; Blue outline = Male; number on the bat = Number of flies released on each bat; Coloured in bats = Bats inoculated with Marburg virus; Not coloured in bats = Bats mock inoculated.

Bats were subdivided into six cages (C1–C6). C1–C3 and C6 contained four MARV-inoculated and two contact control bats each, C4–5 contained six and five MARV-inoculated bats, respectively. All bats in C1–C5 were artificially infested with flies collected at Matlapitsi cave as described in Section 2.3 and released on bats within 8–10 h after collection in the field. Each bat was infested with the equal numbers of female and male flies. Bats in C6 were not infested with flies and served as controls for monitoring the potential horizontal transmission of MARV from infected to ectoparasite-free, in-contact bats. The sex of the experimental animals in each cage, the inoculation status, and the number of flies released on bats is given in Figure 6.

Experiment II: The aim of this experiment was twofold: (1) to investigate the susceptibility of bat flies to MARV following IC inoculation, (2) to attempt the transmission of MARV from IC-inoculated flies to MARV-seronegative bats.

**Figure 7:**
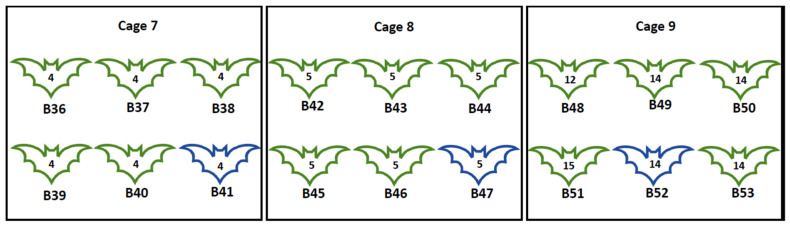
Experiment II. Number, sex of bats, and the number of bat MARV-inoculated flies released on seronegative Egyptian rousette bats. B = Bat identification number 36–53; Green outline = Female; Blue outline = Male; number on the bat = Number of MARV-inoculated flies released on each bat.

**Figure 8:**
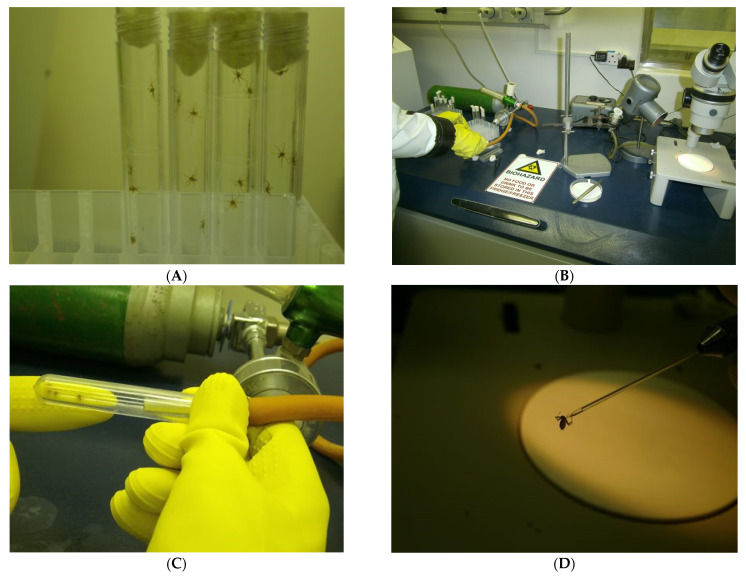

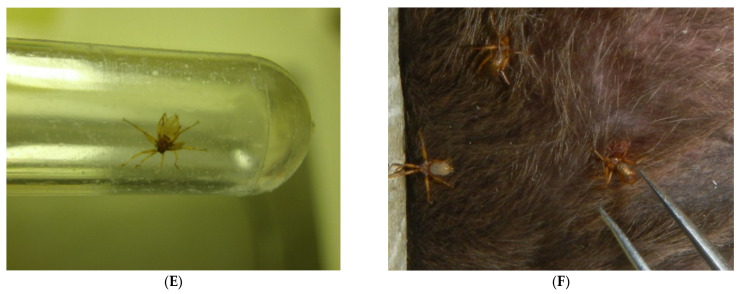
Bat flies kept in plastic tubes for anaesthesia (**A**); Stereomicroscope with light source, inoculation equipment, and CO_2_ cylinder (**B**). Anaesthesia of flies with CO_2_ (**C**); Intra-coelomic inoculation of flies with Marburg virus (MARV) under stereomicroscope (**D**); Inoculated female bat fly in a glass tube recovering from anaesthesia and inoculation (**E**). Release of inoculated flies on MARV-seronegative bats (**F**).

As for experiment I, 18 MARV-seronegative bats aged 8–12 months were moved from the flight cage in BSL3 to the BSL4 animal containment room and placed in three cages (C7–C9). After acclimatisation for one week in BSL4, all 18 bats were artificially infested with IC-inoculated female flies collected at Matlapitsi Cave, as described in Section 2.3. After anaesthesia with CO_2_, flies were IC inoculated under a stereomicroscope with 0.34 ± 0.02 μL inoculum containing 10^5.3^ TCID_50_/mL of MARV (68 ± 4 TCID_50/_fly). Glass needles for inoculation were made by mechanically drawing Pyrex capillary tubes to a fine point using a Leitz needle puller. Following inoculation, flies were temporarily placed in individual tubes for recovery before release onto bats. The sex and numbers of experimental ERBs in each cage, and the number of IC MARV-inoculated flies released on bats are given in Figure 7. The experimental procedures used for experiment II, including flies’ anaesthesia, inoculation, and the release of inoculated flies on MARV-seronegative bats, are shown in Figure 8. In addition, the release of inoculated flies on bats in BSL4 is shown in the video supplementary material.

The possibility of MARV vertical transmission by bat flies was also investigated in this study by testing F1 pupae and F1 flies collected in experiments I and II.

All animals were anaesthetised prior to inoculation and specimen collection as previously described [28]. Blood for serological and molecular testing was taken at regular intervals by either cephalic vein or cardiac puncture, and it was processed as previously described [28]. At termination of the experiments, animals were euthanised under deep anaesthesia by exsanguination via cardiac puncture [28]. All efforts were made to minimise distress. Experiment I was terminated 43 days post SC inoculation and experiment II was terminated 38 days post IC inoculation. All bats were monitored for viremia, seroconversion, food uptake, behavioural changes, and clinical symptoms. Count of adult flies, F1 pupae, and F1 flies was done visually and when flies and pupae were collected at regular intervals during experiments. Flies were collected into individual microtubes, and pupae were collected in pools (3–4 per tube) and processed as described in Section 2.5.

### 2.8. Serology

An indirect enzyme-linked immunosorbent assay (I-ELISA) based on purified recombinant MARV (Musoke) glycoprotein GP antigen (recGP I-ELISA) (Integrated BioTherapeutics, Gaithersburg, MD, USA) was used for the detection of anti-MARV IgG antibody in bat sera using a previously described procedure [29]. Briefly, ELISA plates were coated with 100 μL/well of stock antigen (0.6 mg/mL) diluted 1:1500 in PBS pH 7.2 and incubated overnight at 4 °C. Plates were washed three times with PBS pH 7.2 containing 0.1% Tween-20. The same washing procedure followed each subsequent stage of the assay. Then, the coated plates were blocked with 10% fat-free milk powder in PBS and incubated for 1 h at 37 °C. After washing, 100 μL/well of the control and test sera diluted 1:100 in PBS containing 2% milk powder was added to the plates. After 1 h incubation at 37 °C, plates were washed, and 100 μL of a 1:2000 dilution of the anti-bat immunoglobulin–horseradish peroxidase conjugate (Bethyl, Montgomery, AL, USA) was added to each well. After incubation for 1 h at 37 °C, plates were washed, and 100 μL of 2,2′-azino di-ethyl-benzothiazoline-sulfonic acid substrate was added to each well. Plates were incubated in the dark for 30 min at room temperature. Reactions were stopped by the addition of 100 μL/well of 1% a sodium dodecyl sulphate solution, and optical densities (OD) were measured at 405 nm. The mean OD readings were converted to a percentage of positive (PP) control serum and the cut-off value of ≥16.78 PP was used for the interpretations of recGP I-ELISA results [29].

### 2.9. Real-Time Quantitative Reverse-Transcription Polymerase Chain Reaction and Virus Isolation

We used real-time quantitative reverse transcription polymerase chain reaction (qRT-PCR) as described previously [28]. Samples with cycle threshold values ≤ 40 were regarded as positive. RNA copy numbers detected in samples were converted into median tissue culture infectious dose (TCID_50_) genome equivalent [28].

Virus isolation attempted on field-collected bat flies was performed as previously described [75]. Briefly, the wells of 24-well tissue culture plates (Nunc, Roskidle, Denmark) were seeded with Vero E6 cells and grown to 80–90% confluency in Eagle’s minimum essential medium (EMEM, Lonza, Basel, Switzeraland) supplemented with antibiotics (Penicillin/Streptomycin/AmphotericinB, Lonza, Basel, Switzerland) and 10% foetal calf serum at 37 °C and 5% CO_2_. Culture medium was removed, and the monolayers in individual wells were inoculated with 200 µL of ectoparasite homogenate. After one hour adsorption at 37 °C, the inoculum was removed, and fresh EMEM containing antibiotics and 2% foetal calf serum added. The 24-well plates were incubated for 14 days, and cytopathic effects (CPE) were monitored. A second and third blind passage was performed for all samples by inoculating and incubating monolayers as described above with 200 µL of undiluted supernatant from the preceding passage.

### 2.10. Statistical Analysis

The statistical differences in mean viral load levels in flies fed on MARV-infected bats and in MARV-inoculated flies were calculated using a non-parametric, two-sample Wilcoxon rank (Mann–Whitney) test because of not normal data distribution. Statistical differences in bat fly survival were calculated using Fisher’s exact test. Statistical analysis was done using Stata 13 (StataCorp, College Station, TX, USA) and Microsoft Excel (2016). For both analyses, a *p*-value ≤ 0.05 indicates a statistical significant difference.

## 3. Results

### 3.1. Estimation of Infestation Rate of Egyptian Rousette Bats by Bat Flies

Estimates of infestation rates and the number of bat flies found on ERBs in Matlapitsi Cave were calculated based on two separate collections in November 2016 are given in Table 1. Irrespective of the collection date, sexes, and physiological status of bats, infestation rates by *E. africana* flies were very high, ranging from 95.2% to 100%. An average number of flies per bat ranged from 7.0 ± 6.9 to 27.3 ± 24.1. Infestation was higher on male ERBs, and up to 46 to 67 bat flies could be collected from a single male (Table 1). Despite rather heavy mean infestation rate per bat, all bats sampled appeared healthy.

### 3.2. Field Infection Rate with Marburg Virus in Nycteribiid Bat Flies

A total of 1653 bat flies collected from ERBs roosting at Matlapitsi Cave from June 2012 to July 2018 were tested for the presence of MARV. Virus isolation was attempted on 273 individual flies collected from March 2013 to March 2014, and the remaining flies were tested by qRT-PCR. Of 1693 bat flies tested, only one (0.06%; Ct value = 38.71), from a single night’s collection of 315 specimens (0.3%) in April 2016, tested positive for the presence of MARV RNA. From 273 flies subjected to virus isolation, 13 isolates were obtained, of which two were identified as a novel fusogenic orthoreovirus and 11 isolates were identified as a novel orthobunyavirus; these findings were reported elsewhere [75,76].

### 3.3. Survival and Biology of Bat Flies

Of the 626 field-collected bat flies, 68 (10.9%) died between the time of collection and the time of their release on captive-bred bats in BSL4 (within approximately 8–10 h, including about 5 h of road transportation). Dead bat flies were collected into individual microtubes and stored at −70 °C until testing. An additional 15 bat flies died after IC inoculation and were discarded. A total of 400 bat flies were released on bats in experiment I (C1–C5; n = 400), and 137 bat flies were released on bats in experiment II (C7–C9); 6 IC-inoculated bat flies were immediately stored −70 °C after inoculation until testing. During the time of experimentation, the average percentage of RH in two animal isolators housing bat cages was 41.6 ± 16.3 SD (mean ± standard deviation) and 35.9 ± 36.2, while the average temperature was 20.2 ± 0.95 and 21.1 ± 2, respectively. The average measurements of humidity and ambient temperatures in animal isolators were within those recorded in Matlapitsi Cave.

The survival rates and the number of bat flies collected for qRT-PCR testing after the release of flies on infected bats and after the release of IC-inoculated bat flies on MARV-seronegative bats are given in Table 2. About 50% of bat flies fed on infected bats and IC-inoculated bat flies were alive on day 5 PI, and about 15% were alive on day 22 PI, respectively (Table 2). The survival rates were not significantly different for flies fed on infected bats and for IC-inoculated flies fed on MARV-seronegative bats, on days 3 (*p* = 0.878), 12 (*p* = 0.898), 15 (*p* = 0.730), and 22 (*p* = 0.674) PI (Table 2). There was a significant decrease in the number of flies in both experiments between days 3 and 12 PI (*p* < 0.001), but there was no significant decrease between day 12 and 15 PI (*p* = 0.554 and *p* = 0.254) and day 15 and 22 PI (*p* = 0.317 and *p* = 0.462).

While we did not mark flies to monitor their movement between bats within the same cage, judging from the changing infestation numbers observed on individual bats on a daily basis and due to the close roosting and social behaviour of the bats, it can be assumed that active movement was taking place.

**Figure 9:**
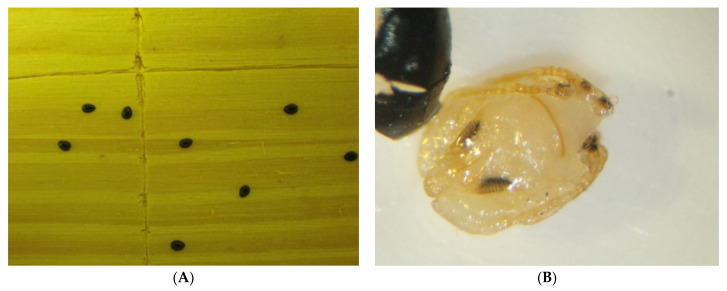
Bat fly pupae deposited on wooden plank (**A**). Emerging bat fly (**B**).

F1 pupae were deposited regularly (Figure 9), with the first seen the day after release of flies in C1–C8. Most pupae were deposited on the wooden roosting substrate, but occasionally, they were also deposited on the walls of cages. Over a period of 43 days of experiment I, 216 pupae were counted, of which 96 were collected for testing (Table 3). Over a period of 38 days of experiment II, 179 pupae were counted, of which 85 were collected for testing (Table 3). The first emergence of F1 bat flies was noted in C1–C8 around 22 days PI. Of the 120 F1 emerged flies, 100 were collected for testing (Table 3).

### 3.4. Horizontal Transmission

#### 3.4.1. Oral Susceptibility of Bat Flies to MARV following Feeding on Viremic Bats and Attempted Transmission of MARV by Bat Flies from Viremic Bats to in Contact Bats

**Figure 10:**
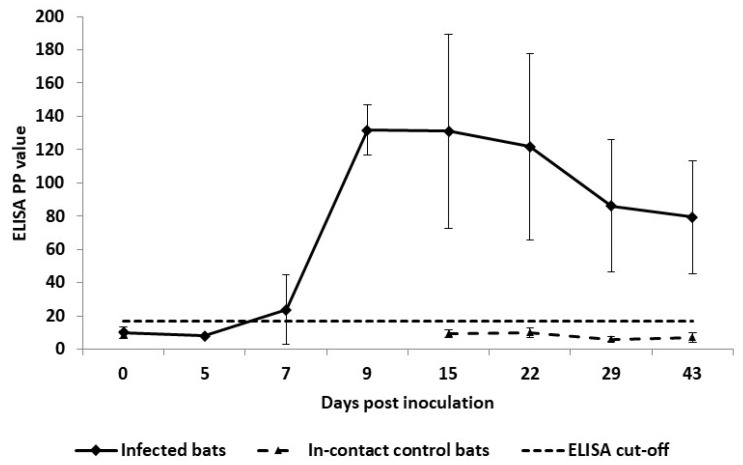
Mean IgG antibody levels (with standard deviations) in Egyptian fruit bats inoculated subcutaneously with Marburg virus (MARV) and in-contact MARV-seronegative bats artificially infested with bat flies. Results for anti-MARV IgG antibody by enzyme-linked immunosorbent assay (ELISA) are shown as the percentage positivity (PP) relative to positive internal control serum; the dotted line without markers denotes an ELISA cut-off value of 16.78 PP [28].

All bats remained clinically well, maintained normal food uptake, and no abnormal behavioural or clinical disease were recorded. None of the bats sustained apparent injury during the duration of the experiments. The first seroconversions were detected on day 7 after SC inoculation, and by day 15, all the infected bats in C1–C6 became MARV-seropositive. The highest levels of anti-MARV IgG level were recorded 2–3 weeks after SC inoculation, which was followed by a decrease on day 29. None of the eight in-contact control bats in C1–C3, and C6, seroconverted (Figure 10).

All bats in C1–C6 became viremic within 3–5 days post inoculation (PI), with a mean (±SD) peak viremia of 10^4.6±0.61^ TCID_50_/mL on day 5 PI; most bats cleared detectable viremia by day 9 PI (Table 4). At the peak of viremias, there was no statistical difference (*p* = 0.260) in their levels between MARV-infected/infested bats and MARV-infected non-infested bats. Viremia levels in individual bats ranged from 10^1.84^ (day 9 PI) to 10^5.0^ (day 5 PI) TCID_50_/mL.

We did not determine the blood meal size of bat flies in our study, and this information is not available in the literature. However, engorged flies with visible blood were seen on bats and during their collection by the naked eye. The size of adult *E. africana* is 2.5–3.0 mm, of which one-third constitutes the abdomen [80]. Assuming the latter is approximately half a sphere, the volume of the abdomen would be no more than 2.1 mm^3^ using the following formula: 2/3∏r^3^.

Various species of hematophagous arthropods can ingest a blood meal of up to one to three times their body size. For example, Zeng et al. [81] estimated that the mean (±SD) size of a blood meal ingested by *Ornithodoros turicata* nymphs is 31.5 ± 16.8 mg, representing a 3.5-fold increase from their respective unfed weights of 12.6 ± 2.5 mg. Adult male *Glossina morsitans* with an estimated mean body weight of 22.0 ± 2.2 mg [82] ingest mean blood meal size of 26.1 ± 0.9 mg [83]. Males and females of unfed pigeon fly (*Pseudolynchia canariensis*) with a mean body weight of 4.80 ± 0.13 mg and 5.03 ± 0.94 mg can take on average 6.5 mg and 5.5 mg of blood meal, respectively [84]. However, the range of blood meal size can vary substantially, depending on the individual flies [81,82,83,84]. *Pseudolynchia canariensis* is approximately two times bigger than *E. africana*. Taking into account this information and the estimated size of *E. africana*’s abdomen, we extrapolate that the average size of blood meal volume for this species would be ≈3 µL, corresponding to ≈3 mg. We use the estimated blood meal size for the interpretation of virological results in bat flies fed on viremic bats.

The mean viral load in bat flies fed on viremic bats ranged from 10^0.25±0.08^ to 10^2.01±1.24^ TCID_50_/fly (Table 4), with a concentration of MARV per individual fly ranging from 10^0.16^ to 10^3.32^. There was a significant statistical difference (*p* = 0.042) between viral loads recorded in flies on day 5 and day 7 PI with mean viral load in flies on day 7 PI being 8 × higher compared to that on day 5 PI (Table 4). Flies with mean viral loads ≥ 10^2.0^ would have to ingest ≥ 1 µL of viremic blood having ≥ 10^5.0^ TCID_50_/_mL_ of the virus. This indicates that in most flies, viral loads represent the amount of the virus taken from viremic bats in the estimated size of the blood meal and thus does not evidence MARV replication. Four (40%) out of 10 flies tested on day seven PI had MARV load >10^3.0^ TCID_50._ Would MARV replicate in those flies, they would have to take >10 µL of ≥10^5.0^ TCID_50_/_mL_ of viremic blood, which is about three times more than the volume of the estimated blood meal size.

Flies feed regularly on their hosts, and the higher concentration of MARV in flies recorded on day 7 PI might be due to accumulation of the virus from the previous blood meals. It could be also that some level of replication was still taking place in the bats’ blood cells upon uptake by flies or/and that we underestimated the blood meal size. Even though some level of transient MARV replication could take place in flies, e.g., the cells of the midgut, following feeding on viremic bats, it did not result in disseminated infection, as evidenced by results in flies collected from bats on days 9–43 PI; all tested negative for MARV RNA (Table 4).

The lack of susceptibility to oral infection with MARV upon feeding on viremic bats and the incapacity of flies to horizontally vector MARV is supported by the fact that none of the MARV-seronegative in-contact bats developed viremia or seroconverted within 43 days, despite the active movement of flies between infected and control bats.

#### 3.4.2. Susceptibility of Bat Flies to MARV following IC Inoculation and Attempted Transmission of MARV from IC Inoculated Flies to Bats

All bat flies tested on day 0 (immediately after inoculation) and day 7 after IC inoculation were positive for MARV RNA but negative on days 12–38 post IC inoculation (Table 5). There was statistically no significant difference in MARV concentration on days 0 and 7 after IC inoculation (*p* = 0.474), and viral loads were consistent with that of the inoculum. None of the 18 MARV-seronegative bats became viremic or seroconverted within 38 days after infestation with MARV-inoculated flies. 

### 3.5. Vertical Transmission

Of the F1 generation 181 pupae and 100 flies collected during experiments I and II (Table 3), all tested negative by RT-PCR, which indicates the biological incapacity of bat flies to transmit MARV vertically.

## 4. Discussion

Understanding of filoviruses maintenance in natural host systems is important in defining the mechanisms of their transmission among reservoirs and those triggering spillover outside their primary ecological habitats. This knowledge will help develop science-based risk reduction strategies and public health messaging. Experimental studies seem to confirm that clinical, virological, and immunological responses are consistent with ERB being a reservoir host for MARV [28,29,30,31,32,33]. Several portals of entry of MARV have been postulated. Experimental SC inoculation of ERBs with MARV, as a proxy for mechanical transmission through skin injury, has been shown to lead to disseminated infection of alimentary, respiratory, and reproductive organs [29,30], but horizontal or vertical transmission resulting from this route of infection has not yet been confirmed. The unsuccessful infection of ERBs with MARV via the oronasal route [28] and absence of aerosol transmission or infected nasal secretions, despite the presence of low levels of MARV in ERB lungs [29], argues against a primary role for transmission via the respiratory route.

Currently available experimental data point to the possibility of oral and/or faecal route of MARV transmission [29,30,31,32], which could explain why primary human MVD cases are often associated with bat habitats in the absence of direct contact through bites, collisions, or scratches [2]. While Schuh et al. [32] seems to demonstrate in-contact bat-to-bat transmission, the delay in viremia and seroconversion in naïve contact ERBs is difficult to explain. Persistent infection in one or more of the infected bats might be one explanation, but the mechanisms that presumably triggered virus reactivation and acute infection after a prolonged incubation period would require further studies. The postulated prolonged MARV incubation period of at least 7 months in ERBs following horizontal transmission of the virus [32] seems to be not supported by findings in ERBs under natural settings. Namely, not only there is a rapid increase in MARV seropositivity in juvenile bats shortly after the waning of maternal immunity [39,74], but shedding of the virus in saliva and faeces is very low [74]. These results suggest that faecal–oral bat-to-bat transmission might be not a sustainable mechanism for the maintenance and perpetuation of marburgviruses. Furthermore, MARV inoculation into the oral cavities of naïve ERBs did not result in infection [28].

Blood-sucking flies of the families Nycteribiidae and Streblidae (Diptera: Hippoboscoidea) represent ectoparasites that are highly associated with bats (59–62, 78, 80). Their role in the ecology of filoviruses has been postulated for a long time [55] but never studied experimentally. To date, nearly 40 species of nycteribiid and 32 of streblid bat flies have been documented in Africa [78,85]. However, knowledge of bat flies that parasitise cave-dwelling chiropterans is limited. Entomological surveys conducted in Gabon demonstrated that the bat fly species *Nycteribia schmidlii scotti* and *Penicillidia fulvida* were strongly associated with *M. inflatus*, while *E. africana* was preferentially associated with *R. aegyptiacus* [68]. Infestation rates for ERBs in this study varied significantly throughout the year and between caves (from 29.4 to 91.5%), but the mean number of *E. africana* flies per infected bat (2.66) remained stable [68]. Nycteribiid host preference was demonstrated in Madagascar and the Comoros, where *E. madascarensis* and *E. theodori* were specifically associated with local frugivorous *Rousettus* species [59,62]. In our study, both infestation rates of ERB’s Matlapitsi population as well as the mean number of *E. africana* flies per bat were higher than those reported from Gabon [68]. Bat species that are roosting in more permanent, enclosed structures, such as Mahune Cave, are more likely to carry heavier parasite loads and to harbour more species of flies [86].

The detection of significant viremia in experimentally infected ERBs, host-specificity of the haematophagous nycteribiids for ERBs, and their ability to take multiple blood feeds make a strong case for a natural, feral transmission cycle for MARV. All that is still required is the development of a MARV infection in the midgut and haemocoel of the bat fly, with dissemination to the salivary glands and into the saliva for successful transmission to receptive bats. Recently, viruses other than marburgviruses have been isolated from nycteribiids, including *E. africana* [67,75,76], demonstrating their potential in vectoring pathogens.

The successful infection of a hematophagous insect by a pathogen and its subsequent replication and dissemination to enable per-oral transmission during feeding is dependent on a number of factors including the vector genotype, environment, and viral pathogen, which are all interlinked and affect vector competence and vector capacity [87]. Vector competence is the ability of a vector to support pathogen infection, replication, dissemination, and transmission [88]. Vectorial capacity refers to the ability of the vector population to transmit pathogen, and it incorporates host preference, biting rates, survivorship, vector competence, and extrinsic incubation period (time from ingestion to transmission) that are subjected to environmental modifications [88]. One of the most important factors affecting vectorial capacity is the temperature, which impacts on immature vector developmental stages, the number of generation produced, and consequently the size of the adult population [89]. Temperature also significantly affects extrinsic incubation and virogenesis [88,89,90,91]. A number of genetically driven factors such as innate immunity and tissue replication and escape barriers need to be overcome by an arbovirus, including the midgut infection barrier, midgut escape barrier, salivary gland infection barrier, and salivary gland escape barrier. Genetic factors influencing vector competence can vary widely between vector species, and only specific arbovirus species are adapted to overcome these barriers successfully [89,92]. Hardy et al. [93] proposed a number of steps for a productive arbovirus infection to occur in a vector, including the initiation of infection in the midgut, dissemination from the midgut to secondary tissues, infection of salivary glands (and sometimes reproductive tissues for vertical transmission to offspring), and finally release of the virus into saliva, which is a source of horizontal transmission to an uninfected vertebrate host. While all these variables could not be investigated in our study, the results obtained strongly suggest that bat flies do not support MARV replication, both following the natural ingestion of infectious viremic blood and following an artificial inoculation.

Relatively little is known about the life history and reproductive biology of bat flies, and detailed information regarding the life cycle of *E. africana*, specifically, is not available. Nonetheless, the characteristics of the life cycle and breeding biology of *E. africana* observed in our study generally resembles what is currently known. These include the following: (1) deposition site (within the confines of the bat’s roosting environment); (2) deposition of a single pupa on the substrate; (3) 3–4 weeks (average 23 days) for the emergence of teneral adults from pupa; (4) locating and colonising a host by newly emerged flies; and (5) time interval between successive depositions of pupae of about 10 days [94,95,96]. Due to the time limitation of our experiment and sampling schedule, the exact life span of *E. africana* could not be determined, but it appears to correspond to that estimated for other species of bat flies, *E. sundaica* and *Basilia hispida* [95,97].

The mean temperature ranges during the housing of bats and ectoparasites under BSL4 environmental conditions were within those recorded at Mahune Cave where we sourced both bats and ectoparasites for this study, but the mean percentage of RH was lower. The environment of an ectoparasite extends from its immediate environment such as the host itself to the surrounding ecosystem in which the host lives, including anthropogenic and climatic conditions [98]. The roosting biology of bats influences the quality and quantity of parasitism by bat flies [99,100,101], which in turn might influence the likelihood and capacity of vectoring pathogens. From the point of vertical transmission, it should be noted that pupation takes place off the host, in the bat’s roost, and it lasts a few weeks. This means that bat flies spend possibly up to one-third of their life off the host and in the immediate roosting environment, mainly as pupae [101]. During this time, both the nature of the roosting substrate and temperature might influence the replication of a pathogen. These variables and resulting potential limitations could not be addressed, but the full life cycle of *E. africana* was observed in our study. This indicates that the provided environmental conditions suitably mimicked natural settings, and thus, the results obtained likely reflect on the true inability of flies to support MARV replication and consequently the lack of capacity for either horizontal or vertical transmission.

Of 1693 field-collected bat flies, one was positive by qRT PCR but, interestingly, this fly was from a single night collection in April 2016 of 315 specimens at Matlapitsi Cave in South Africa. While the very low field infection rate seems to additionally support experimental findings that bat flies do not play a significant role in the transmission and enzootic maintenance of MARV, our study was not designed to determine the prevalence of MARV infection in the Matlapitsi nycteribiid population. However, it is worth noting that the one positive specimen was collected during the time of the year that coincides with a significantly higher seasonal transmission of MARV in the Matlapitsi ERB population [39,74].

The detection of MARV in bat flies in our study, following feeding on viremic bats up to 7 days PI, might have important ramifications in the context of the postulated oral mechanism of MARV transmission [30,32]. Although peak viremias in both sylvan and experimentally infected bats tend to be rather low and of moderate duration [28,29,30,31,32], the high consumption rates of ectoparasites [102] coupled with the high density and roosting behaviour of *Rousettus* bats, regular, multiple blood feeds by bat flies, and the extent of time spent by bat flies on ERBs might result in increased exposure to the virus. In has been estimated that the mean number of nycteribiid flies consumed by an adult Madagascar fruit bat (*Rousettus madagascariensis*) during auto- and allo-grooming was 37 daily. When this number was extrapolated on the estimated number of adult *Rousettus* bats at the roost site in the Grotte des Chauves-souris, d’Ankarana National Park in Madagascar, the projected daily consumption rate was 57,905 ectoparasites daily [102]. The consumption of contaminated ectoparasites could provide the mechanism for oral MARV transmission. In addition, virus transfer might also take place through the contamination of skin and mucosal membranes when contaminated ectoparasites are squashed during grooming (scratching or licking the body and wing phalanges), eating, biting or fighting, and via contaminated mouthparts during feeding.

In the present study, attempts to infect bat flies by feeding on viremic bats and by artificial inoculation were unsuccessful. However, one has to consider some of the limitations of this first vector competence study, including the potential impact of the dose of inoculum used, the source of the virus, environmental conditions, and the numbers of flies tested. The results from this preliminary study seem to demonstrate that bat flies do not act as biological vectors of MARV. However, considering that bat flies can carry contaminated blood following feeding on viremic bats, their potential role in mechanical transmission cannot be ruled out and would require further investigation. The eating of flies engorged with MARV-contaminated blood by bats with abrasions of their oral cavities—or direct contact with other damaged mucous membranes and/or skin with contaminated flies’ blood or indirectly via contaminated fomites—might result in the inoculation of MARV. The inoculation of ERBs via the SC route results in MARV replication in various tissues [28,29,30,31,32,33]. In our study, none of the bats sustained apparent mucous membranes and/or skin injury or suffered due to mucous and skin disorders during the experiments. However, biting and fighting among bats resulting in mucous membrane and skin injury are observed under natural settings [29].

The Matlapitsi Cave in South Africa is one of many African ecological niches for highly biodiverse mammals and ectoparasites. The multispecies roosting behaviour with potentially highly specific host–parasite interactions might govern transmission cycles of bat fly-associated pathogens. Understanding these networks requires systematic and long-term investigations. To our knowledge, our experimental vector competence work described here is the first to demonstrate the successful colonisation and breeding of bat flies on ERBs and IC inoculation of bat flies under BSL4 conditions. The host–parasite model established here constitutes a potentially valuable research resource for investigating the role of bat ectoparasites in transmitting zoonotic pathogens of public health importance.

## Figures and Tables

**Table 1:** Infestation rate of Egyptian rousette bats by bat flies at Matlapitsi Cave estimated on two different collection dates in November 2016, South Africa.

Date of Capture and Flies Count	No. Bats Captured	No. ERB Females	No. ERB Males	No. Non-Pregnant Females without Pups	No. Pregnant Females	No. Females with Pups
2–3 November 2016						
Total no. bats captured	56	51	5	- ^a^	-	-
Total no. flies	551	461	90	-	-	-
Infestation rate (%)	98.3	98.0	100	-	-	-
Mean ± SD ^b^ no. flies/bat	9.8 ± 8.3	9.0 ± 6.8	18.0 ± 16.6	-	-	-
Range in no. flies/bat	0–46	0–28	5–46	-	-	-
14–15 November 2016						
Total no. bats captured	62	55	7	19	14	22
Total no. flies	556	365	191	158	73	134
Infestation rate (%)	95.2	96.4	100	89.5	100	95.5
Mean ± SD no. flies/bat	9.0 ± 12.0	7.0 ± 6.9	27.3 ± 24.1	8.3 ± 10.4	5.2 ± 3.6	6.1 ± 4.0
Range in no. flies/bat	0–67	0–36	1–67	1–36	1–14	1–12

^a^ No count done; ^b^ Standard deviation.

**Table 2:** Survival and number of bat flies collected for laboratory testing (**A**) after feeding flies on MARV-infected bats and (**B**) after feeding of MARV-inoculated flies on R. *aegyptiacus* bats.

**Flies Fed on Infected Bats**	**Cage No.**							**Count/No. Collected** **Survival (%)**
	**1**	**2**		**3**	**4**		**5**	
(**A**)								
Day ^a^ 0	60 ^b^	60		60	120		100	400/0
Day 3	48/1 ^c^	42/2		44/2	nc ^d^/15		nc/13	134/33 134/180 ^e^ (74.4)
Day 5	28/0	29/0		31/0	nc ^e^ /25		nc/20	86/45 93/180 (51.7)
Day 7	nc/0	nc/7		nc/0	nc/15		nc/12	nc/34
Day 9	nc/0	nc/0		nc/0	33/0		27/0	60/0 87/220 (39.5)
Day 12	9/0	12/2		26/4	nc/0		nc/0	47/6 (26.1) 47/180
Day 15	8/3	7/3		10/3	nc/8		nc/4	25/21 31/180 (17.2)
Day 22	2/2	4/0		8/0	12/6		9/4	35/12 56/400 (14.0)
Day 29	0	2/2		5/5	8/8		2/2	17/17 29/400 (7.3)
**Inoculated Flies Fed on Bats**	**Cage No.**							**Count/No. Collected** **Survival (%)**
	**7**		**8**			**9**		
(**B**)								
Day ^f^ 0	24 ^b^		30			83		137
Day 3	nc/0		nc/0			63/0		63/0 63/83 (75.9)
Day 7	10/1		14/1			43/5		67/7 67/137 (48.9)
Day 12	5/0		8/0			24/0		37/0 37/137 (27.0)
Day 15	nc		nc			16/16		16/16 16/83 (19.3)
Day 22	2/0		3/0			0/0		21/0 21/137 (15.3)

^a^ Day post infection of bats with Marburg virus (MARV); ^b^ No. of bat flies released on day 0 post inoculation in each experimental bat cage; ^c^ No. of bat flies counted and collected for testing; ^d^ Count not done; ^e^ Survival rates on different days post inoculation; ^f^ Day post infection of flies with MARV. Note: Survival rates on different days post inoculation were calculated using the following formula: percentage survival = (no. flies on day of counting plus no. flies collected in preceding day of count/total no. of flies released) × 100. For example, the survival rate of flies in Cages 1–3 on day 5 post inoculation is 51.7%: for day 5, the count of flies is 86, the number of flies collected on preceding count day is 5, the total number of flies released in C1–C3 was 180; thus, (93/180) × 100 = 51.7%. This calculation is based on the arbitrary assumption that at least flies collected during the preceding count would survive until the next count.

**Table 3:** Number of F1 generation pupal and adult bat flies counted and collected for testing in cages housing flies fed on subcutaneously (SC) inoculated bats (C1–C5) with Marburg virus (MARV) and in cages (C6–C8) housing flies administered MARV by intra-coelomic inoculation (IC).

F1 Pupal and Adult Flies										
Day after SC Inoculation Cages 1–5										
	3	5	7	9	12	15	22	29	36–43	Total
No. pupae	13	28	38	37	31	58	34	37	20	216
New	13	15	21	12	6	33	10	3	7	120
Collected	0	11	13	12	6	34	0	0	20	96
No. flies Collected	0	0	0	0	0	0	5 3	16 14	25 22	46 39
Day after Post IC Inoculation Cages 7–9										
	3	5	7	9	12	15	22	29	33–38	
No. pupae	30	55	68	72	64	65	nc ^a^	51	25	179
New	30	25	13	4	5	11	nc	4	2	94
Collected	0	0	0	13	10	9	0	28	25	85
No. flies Collected	0	0	0	0	0	0	15	48 37	17 15	74 61

^a^ Not count done.

**Table 4:** Experiment 1. Quantitative reverse-transcription PCR (RT-PCR) results in captive-bred *Rousettus aegyptiacus* inoculated subcutaneously with Marburg virus (MARV) (**A**) and in bat flies fed on MARV-inoculated bats (**B**).

**Days Post Inoculation ^a^**	**Number of Bats Tested/Positive (% Viremic Bats)**	**Mean log_10_TCID_50_^b^ ± SD ^c^ /mL Blood**
(**A**)		
3	11/13 (84.6)	3.25 ± 0.63
5	11/11 (100)	4.06 ± 0.61
7	4/9 (44.4)	2.05 ± 0.57
9	2/11 (27.3)	1.86 ± 0.03
12	0/12	0
15–22	0/23	0
**Days Post Inoculation ^d^**	**Number Bat Flies Tested/Positive (% Flies Positive)**	**Mean log_10_TCID_50_ ± SD/Fly**
(**B**)		
3	31/0 (0)	
5	25/3 (12.0)	0.25 ± 0.08
7	39/12 (30.8)	2.01 ± 1.24
9	29/0	
12–15	47/0 (0)	
21–29	25/0 (0)	
36–43 ^e^	20/0 (0)	

^a^ Cumulative qRT-PCR results (Experiment I—bat cages C1–C6) in 27 *Rousettus aegyptiacus* bats inoculated subcutaneously with Marburg virus (MARV) on different days post infection; ^b^ MARV RNA copy numbers detected in samples converted into median tissue culture infectious dose (TCID_50_) genome equivalents [28]; ^c^ Standard deviation; ^d^ Cumulative qRT-PCR results (Experiment I–bat cages C1–C5) in bat flies collected form *Rousettus aegyptiacus* bats on different days post infection; ^e^ Including F1generation flies.

**Table 5:** Experiment 2. Quantitative reverse-transcription PCR (RT-PCR) results in bat flies (*Eucampsipoda africana*) inoculated by intra-coelomic administration of Marburg virus (MARV).

Days Post Inoculation ^a^	Number Bat Flies Tested/Positive (% Positive)	Mean log_10_TCID_50_^b^ ± SD ^c^/Fly
0	6/6 (100)	1.7 ± 0.54
7	10/10 (100)	1.35 ± 1.06
12	16 ^d^/0 (0)	
21	10/0 (0)	
31–38 ^e^	100/0 (0)	

^a^ Cumulative qRT-PCR results in bat flies inoculated by intra-coelomic administration of Marburg virus (MARV); ^b^ MARV RNA copy numbers detected in samples converted into median tissue culture infectious dose (TCID_50_) genome equivalents [28]; ^c^ Standard deviation; ^d^ Of 16 bat flies collected on 16 dpi, six were dissected, and their heads, thoraxes, abdomen, and legs were tested individually; ^e^ Including F1generation of flies.

## Data Availability

The data presented in this study are available within the article.

## Supplementary Materials

The following are available online at https://www.mdpi.com/article/10.3390/v13112226/s1, Video S1: The release of inoculated flies on bats in BSL4.
